# Salt stress affects the bacterial communities in rhizosphere soil of rice

**DOI:** 10.3389/fmicb.2024.1505368

**Published:** 2024-12-06

**Authors:** Yujie Zhou, Zhizhou He, Qiuyun Lin, Yuehui Lin, Kaiyi Long, Zhenyu Xie, Wei Hu

**Affiliations:** ^1^Tropical Crops Genetic Resources Institute, Chinese Academy of Tropical Agricultural Sciences, Haikou, China; ^2^Key Laboratory of Crop Gene Resources and Germplasm Enhancement in Southern China, Ministry of Agriculture and Rural Affairs, Haikou, China; ^3^Key Laboratory of Tropical Crops Germplasm Resources Genetic Improvement and Innovation of Hainan Province, Haikou, China

**Keywords:** *Oryza sativa* L., salt stress, rhizosphere soil, bacterial community, 16S rRNA

## Abstract

Salt is a primary factor limiting the utilization of saline lands in coastal beach areas, with rhizosphere microorganisms playing a crucial role in enhancing crop stress resistance and exhibiting high sensitivity to environmental changes. Rice (*Oryza sativa* L.) is the preferred crop for reclaiming salinized soils. This study determined the microbial communities in rhizosphere soil of rice under different salt stress treatments by high-throughput sequencing. We found that salt stress changed the bacterial community diversity, structure and function in rhizosphere soil of rice. Salt stress significantly reduced the richness and diversity of bacterial communities in rhizosphere soil of rice. The bacterial community was characterized by higher abundance of the phyla Chloroflexi, Proteobacteria and Actinobacteria; the relative abundances of Firmicutes, Acidobacteriota and Myxococcota were decreased, while Bacteroidota and Cyanobacteria were increased under salt stress. The functions of bacterial communities in rhizosphere soil of rice mainly include chemoheterotrophy, aerobic_chemoheterotrophy, phototrophy etc., chemoheterotrophy and aerobic_chemoheterotrophy were significantly higher NS3 (adding 3‰ NaCl solution to the base soil) treatment than NS6 (adding 6‰ NaCl solution to the base soil) treatment. These findings provide a theoretical foundation for the development of specialized salt-tolerant microbial agents for rice cultivation and offer a viable strategy for improving the soil environment of saline coastal lands through the application of beneficial microorganisms.

## 1 Introduction

Soil salinization represents a critical and escalating threat to global agricultural productivity. Presently, approximately 6% of the world’s arable land is affected by salinity, with nearly one-third of irrigated farmland experiencing salinization (Zhai et al., 2023). Projections suggest that by 2050, up to 50% of arable land may be at significant risk of salinity (Pitman and Läuchli, 2002; Jamil et al., 2011; Islam et al., 2021).

Soil salinization poses a significant and escalating threat to global agricultural productivity by adversely affecting numerous morphological and physiological processes in plants, including nutrient uptake, seed germination, and overall growth. Salt stress is recognized as one of the most formidable abiotic stresses impacting crop development (Ponce et al., 2021). The accumulation of salts in the soil not only restricts plant growth but also leads to substantial reductions in crop yields (Qin et al., 2016). Rice is one of the most widely cultivated food crops globally and a staple for over half of the world’s population (Nam MyungHee et al., 2015), is often selected for the reclamation of salinized lands. However, rice is inherently sensitive to soil salinity, particularly to sodium chloride (NaCl), making salinity a critical environmental factor that adversely affects its growth, and lead to substantial reductions in rice productivity (Guo et al., 2023).

The rhizosphere, being the most active microhabitat in the soil, serves as the primary zone for nutrient acquisition by plants. Rhizosphere microorganisms play a crucial role in the decomposition of organic matter and nutrient cycling, which are essential for plant growth, development, and stress resistance. Consequently, there is growing interest in understanding how rhizospheric conditions influence plant stress tolerance. However, these microbial communities are highly sensitive to environmental fluctuations (Degens et al., 2000; Mendes et al., 2011). Research has demonstrated that salt stress induces significant alterations in the structure and composition of soil microbial communities, leading to imbalances in the proportions of functional, beneficial and harmful bacteria within the rhizosphere (Zhang and Yu, 2001). Specifically, salt stress affects both the type and community composition of rhizosphere microorganisms (Kielak et al., 2008).

Soil salinity exerts profound effects on the activity, diversity, and structural dynamics of microbial communities (Chandrasekaran et al., 2014). Changes in salinity levels can result in the loss or activation of certain bacterial species, further disrupting microbial balance (Rath et al., 2019). For instance, studies have shown that Betaproteobacteria become more prevalent in highly saline layers, while the relative abundances of Alphaproteobacteria and Gammaproteobacteria remain stable despite variations in salinity (Jiang et al., 2007). These findings underscore the necessity for detailed investigations into the effects of salinity on bacterial community dynamics to better understand and mitigate the impacts of soil salinization on agricultural systems. Research suggests that soil salinity has exerts detrimental effects on soil microbial communities (Li et al., 2021). Consequently, to enhance rice adaptation to salt stress, it is imperative to investigate the responses of rhizosphere microorganisms under saline conditions. In recent years, researchers have sought to elucidate the potential contributions of rhizosphere microorganisms to plant salt tolerance, extensively examining both the direct and indirect mechanisms through which these microorganisms enhance plant resilience (Yang et al., 2009; Van Dam and Bouwmeester, 2016). Various microorganisms identified in rice under salt stress conditions have been shown to significantly bolster the plant’s ability to withstand salinity, demonstrating notable growth-promoting effects and substantial application potential (Gu et al., 2024). Examples of such beneficial microorganisms include *Bacillus* spp. (Misra et al., 2017), *Microbacterium ginsengiterrae* (Qi et al., 2023), *Pseudomonas punonensis*, and *Chryseobacterium taeanense* (Ma et al., 2024). These findings underscore the critical role of rhizosphere microorganisms in mitigating the adverse impacts of soil salinity on crop performance and highlight their potential in sustainable agricultural practices aimed at enhancing crop resilience and productivity.

In this study, the bacterial community 16S rRNA genes were sequenced using the MiSeq high-throughput sequencing platform. The diversity, structure and function of bacterial communities in the rhizosphere soil of rice were analyzed under varying levels of salt stress. The primary objective was to elucidate the alterations in the rhizosphere bacterial community in response to salt stress and to investigate the relationship between bacterial diversity, structure and function with salinity levels in rice rhizosphere soils. The findings aim to provide a theoretical foundation for the development of specialized salt-tolerant microbial agents for rice cultivation and offer a viable strategy for improving the soil environment of saline lands through the application of beneficial microorganisms.

## 2 Materials and methods

### 2.1 Experimental design and sampling

The study was conducted in the rainproof greenhouses at the Rice Test Base of the Tropical Crops Genetic Resources Institute (109.85°E, 19.83^°^N), Chinese Academy of Tropical Agricultural Sciences, located in Danzhou City, Hainan Province, China. The experimental site belongs to the tropical monsoon climate. Six rice varieties (Reyan 2, Yanxian156, Reke 203, ST002, ST003, and ST0014) were selected for pot experiments from August to November 2023. The experimental soil was sourced from the paddy fields that has been used for rice cultivation in previous years. Each pot is filled with 0.06 cubic meters of dry soil. Three treatments were set up as control treatment (CK, adding fresh water to the base soil, the freshwater used in the experiment had a salinity below 0.2 g/L), 3‰ NaCl stress (NS3, adding 3‰ NaCl solution to the base soil) and 6‰ NaCl stress (NS6, adding 6‰ NaCl solution to the base soil). Each treatment was replicated three times for each variety.

Rice seeds were separately sown in seedling trays according varieties, and transplanted into pots once they reached the four-leaf one-heart stage. Following standard fertilization management for rice, urea and compound fertilizer (N:P:K = 16:16:16) were applied during the experiment. Sixteen plants were maintained per pot (76 cm × 56 cm). A ruler with a consistent scale was inserted into each pot to monitor the water level, maintaining a water layer of approximately 2–3 cm. Salinity was monitored daily using a salinometer, and NaCl solution or fresh water was added as necessary to ensure that the salt concentration in each pot remained relatively stable throughout the experiment. In this experiment, a variation of ±0.5‰ in salt concentration was considered within the normal range.

Salt stress treatment was discontinued 2 weeks before rice maturity and harvest, allowing the soil moisture to naturally dry out. When rice was harvested at maturity, rice plants were carefully excavated to preserve intact root systems. Bulk soil was gently shaken off, and rhizosphere soil adhering to the root surfaces (approximately 2 mm) was collected using sterile brushes. Three plants constituted one replicate. A total of 54 rhizosphere soil samples were collected. Soil samples were transported to the laboratory on dry ice and subsequently stored at −80°C for DNA extraction.

### 2.2 Biological analyses

#### 2.2.1 DNA extraction and PCR amplification

Total genomic DNA was extracted from 54 rhizosphere soil samples using the MJ-soil DNA Extraction Kit (Yuhua Company, China) in accordance with the manufacturer’s instructions. The quality and concentration of the extracted DNA were assessed by 1.0% agarose gel electrophoresis and quantified using a NanoDrop™ 2000 spectrophotometer (Thermo Scientific, United States). The bacterial 16S rRNA genes were amplified using the primer pairs 338F (5′-ACTCCTACGGGAGGCAGCAG-3′) and 806R (5′-GGACTACHVGGGTWTCTAAT-3′) with barcodes. Each sample has three replicates in the PCR amplification process. PCR reactions were conducted with TransStart FastPfu DNA Polymerase (TransGen AP221–02) in a 20 μL reaction volume, comprising 4 μL of 5× FastPfu Buffer, 2 μL of 2.5 mM dNTPs, 0.8 μL. PCR reactions were conducted with TransStart FastPfu DNA Polymerase (TransGen AP221-02) in a 20 μL reaction volume, comprising 4 μL of 5× FastPfu Buffer, 2 μL of 2.5 mM dNTPs, 0.8 μL each of forward and reverse primers (5 μM), 10 ng of template DNA, 0.2 μL of Bovine Serum Albumin (BSA), and ddH₂O to reach the final volume. The amplification protocol included an initial denaturation at 95°C for 3 min, followed by 27 cycles of denaturation at 95°C for 30 s, annealing at 55°C for 30 s, and extension at 72°C for 45 s, culminating in a final extension at 72°C for 10 min and a hold at 10°C until manually terminated. The PCR products of the same sample were purified by extraction from a 2% agarose gel using a DNA gel recovery and purification kit (PCR Clean Up Kit, Yuhua Company, China). Finally, the purified PCR products were quantified using a Qubit™ 4.0 Fluorometer (Thermo Fisher Scientific, USA).

#### 2.2.2 Library preparation and sequencing

Sequencing libraries were subsequently generated using a TruSeq™ DNA Sample Prep Kit. Finally, the cloned libraries were pooled in equimolar amounts and paired-end sequenced on an Illumina HiSeq PE300 platform (Illumina, San Diego, USA).

#### 2.2.3 Data analysis

The resulting high-quality sequences were subsequently clustered into operational taxonomic units (OTUs) at a 97% similarity threshold using Uparse (version 7.0.1090), and chloroplast sequences were excluded from all samples. For each OTU, the most abundant sequence was selected as the representative sequence. Taxonomic classification of these representative sequences was performed using the RDP Classifier (version 2.2) against the Silva 16S rRNA gene database (Release 138).

Alpha diversity indices (Chao, Ace, Shannon, and Simpson) were calculated with Mothur v1.30.1 (Schloss et al., 2009). The Venn diagrams depicting the common and unique Operational Taxonomic Units (OTUs) among the groups were generated using R software (version 3.3.1). Principal coordinate analysis (PCoA) was used to analyze the similarity or difference in sample community composition. To further identify biomarkers distinguishing between groups, Linear Discriminant Analysis (LDA) coupled with LEfSe analysis was conducted (Segata et al., 2011). Predicting the functions of bacterial communities in rhizosphere soil of rice by Functional Annotation of Prokaryotic Taxa (Louca et al., 2016). The Kruskal-Wallis H test was utilized to evaluate statistically significant differences of alpha diversity indices, taxa at the phylum and function in different salt stress treatments. All analysis were performed using the online Majorbio Cloud Platform.

## 3 Results

### 3.1 Overview of sequence and community structure

**Figure 1:**
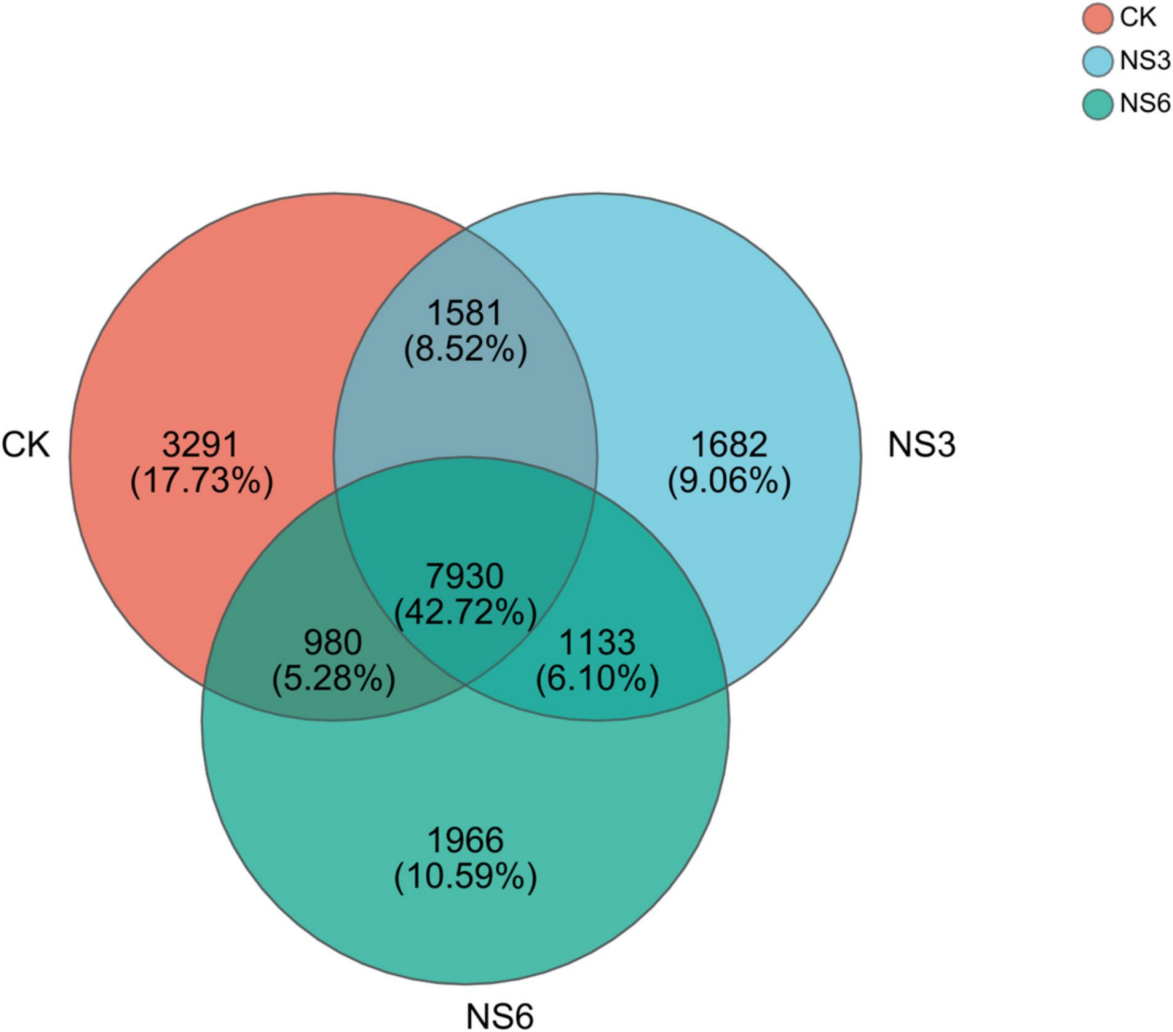
Venn diagram of bacterial OTUs in the rhizosphere soil of rice. CK, control treatment; NS3, 3‰ NaCl stress treatment; NS6, 6‰ NaCl stress treatment.

After quality filtering, a total of 3,713,250 sequences were obtained for the bacterial community analysis. The 18,563 OTUs were identified from sequences with >97% similarity. Specifically, the CK, NS3, and NS6 treatments yielded 13,782, 12,326, and 12,009 OTUs, respectively. Among these, 7,930 OTUs were common to all three groups, accounting for 42.72% of the total OTUs. The unique OTUs were 3,291, 1,682, and 1,966 in the CK, NS3, and NS6 treatments, representing 17.73, 9.06, and 10.59% of the total OTUs, respectively (Figure 1). These results indicate both similarities and differences in the microbial community among the three treatment groups.

**Figure 2:**
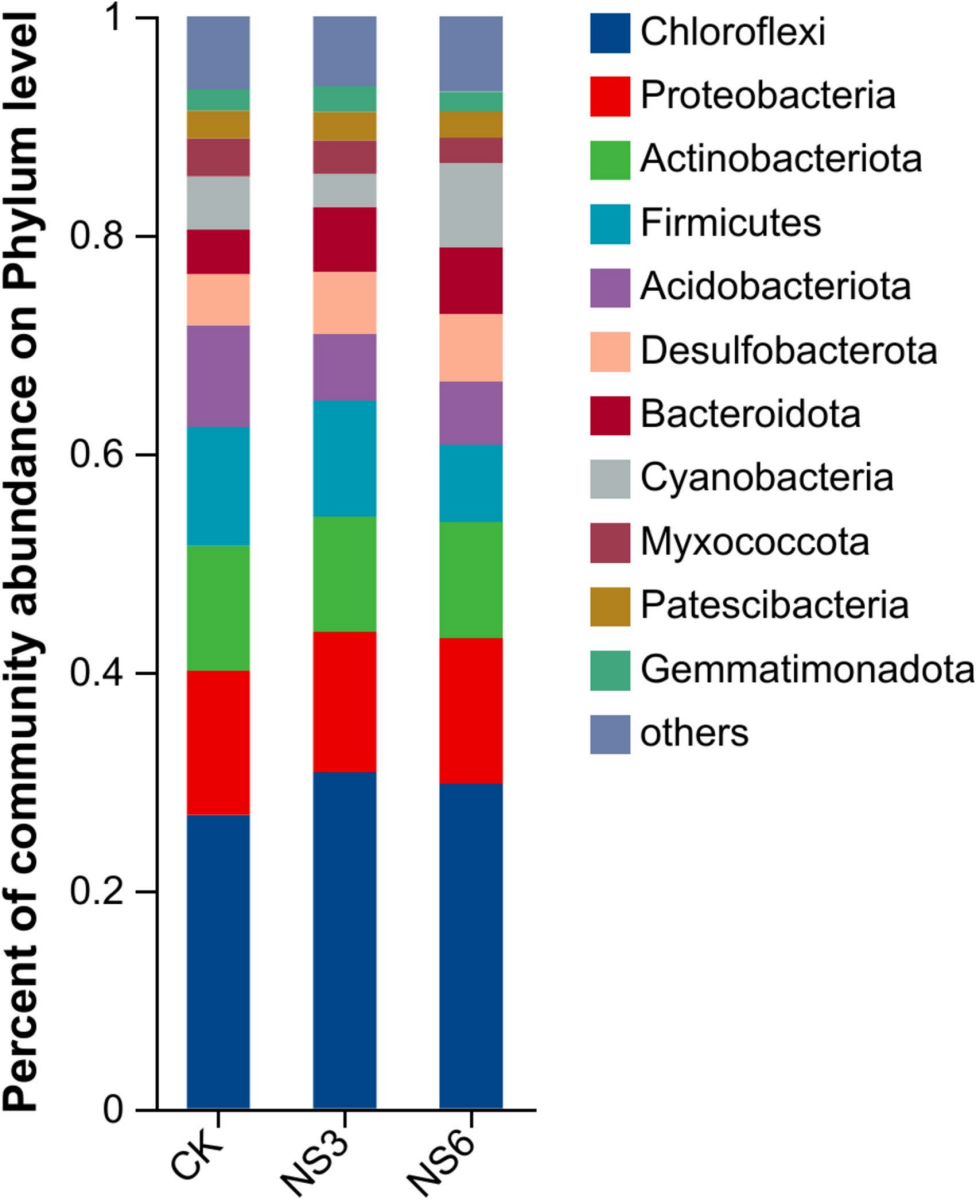
Relative abundance at the phylum level of the bacterial community in rhizosphere soil of rice.

The bacterial sequences were classified into 62 phyla, 194 classes, 475 orders, 802 families, and 1,578 genera. To further elucidate the bacterial community structure, we analyzed the abundance distributions at the phylum and genus levels for each soil group. In terms of relative abundance, Chloroflexi (29.12%), Proteobacteria (13.13%), and Actinobacteria (10.88%) were the dominant phyla. These were followed by Firmicutes, Acidobacteria, Desulfobacterota, Bacteroidota, Cyanobacteria, Myxococcota, Patescibacteria and Gemmatmonadota of each phylum with relative abundances exceeding 2.00% (Figure 2).

**Figure 3:**
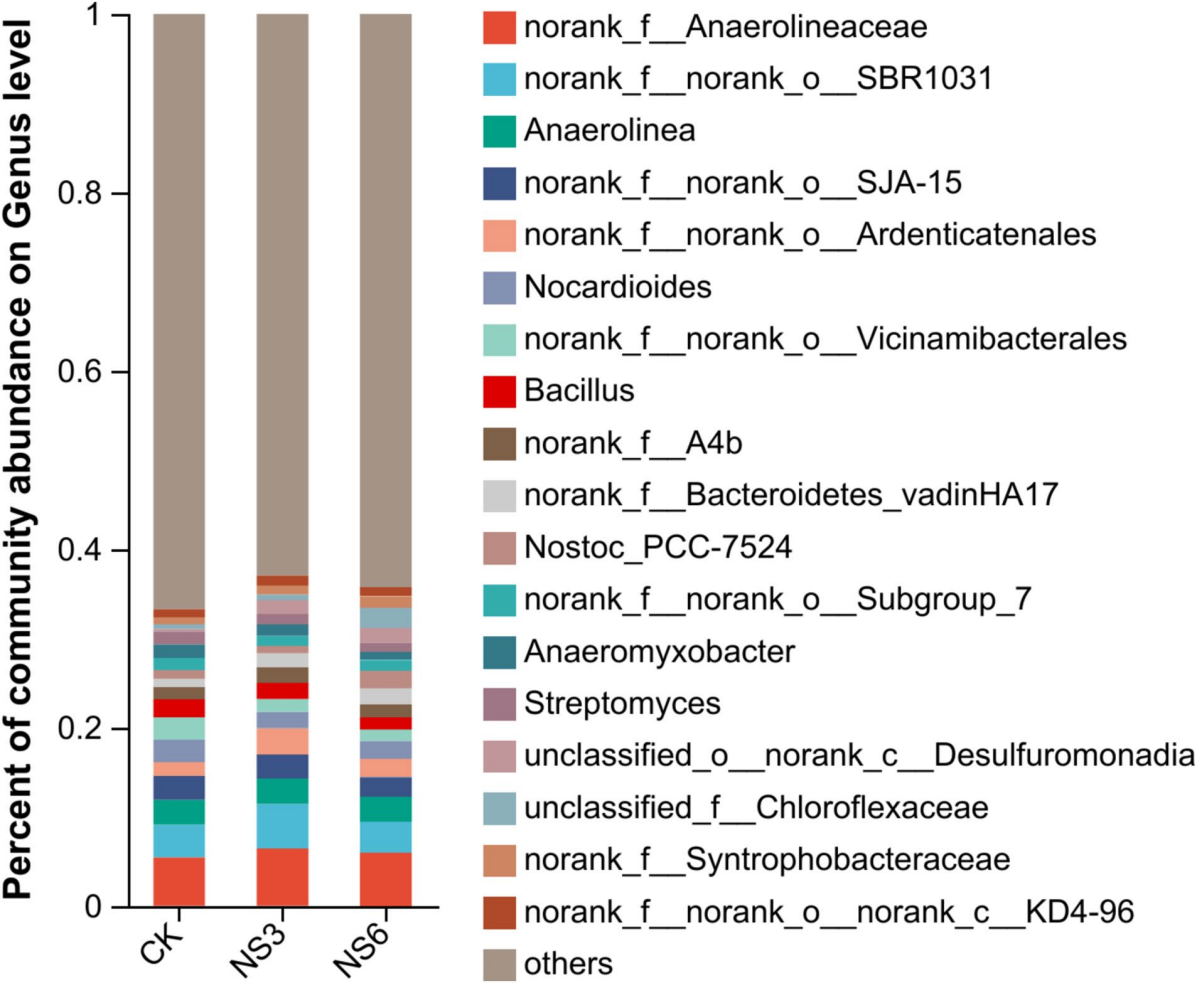
Relative abundance at the genus level of bacterial community in rhizosphere soil of rice.

The genera with relative abundances >1% were depicted in a column chart. The predominant genera identified in the rice rhizosphere soils included *norank_f Anaerolineaceae* (5.92%), *norank_f norank_o SBR1031* (4.06%), *Anaerolinea* (2.81%), *norank_f norank_o SJA* (2.52%), *norank_f norank_o Ardenticatenales* (2.18%), and *Nocardioides* (2.12%), each accounting for more than 2.00% of the relative abundance (Figure 3).

### 3.2 Effects of salt stress on bacterial community diversity

**Figure 4:**
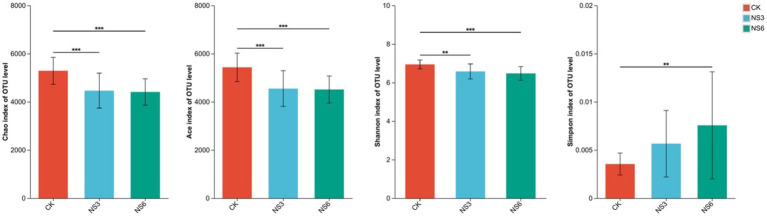
Alpha diversity indices of the bacterial community in rhizosphere soil of rice. CK, control treatment; NS3, 3‰ NaCl stress treatment; NS6, 6‰ NaCl stress treatment.

Alpha diversity analyses were conducted to evaluate the richness and diversity of the microbial communities. The results indicated that the richness indices—Chao, Ace, and Shannon—were significantly higher in the control treatments compared to the 3 and 6‰ NaCl treatments. In contrast, the Simpson index was significantly lower in the control treatments than in the 6‰ NaCl treatments (Figure 4). Based on these alpha diversity indices, it can be inferred that salt stress reduces both the richness and diversity of bacterial communities in the rhizosphere soil of rice.

### 3.3 Effects of salt stress on bacterial community structure

**Figure 5:**
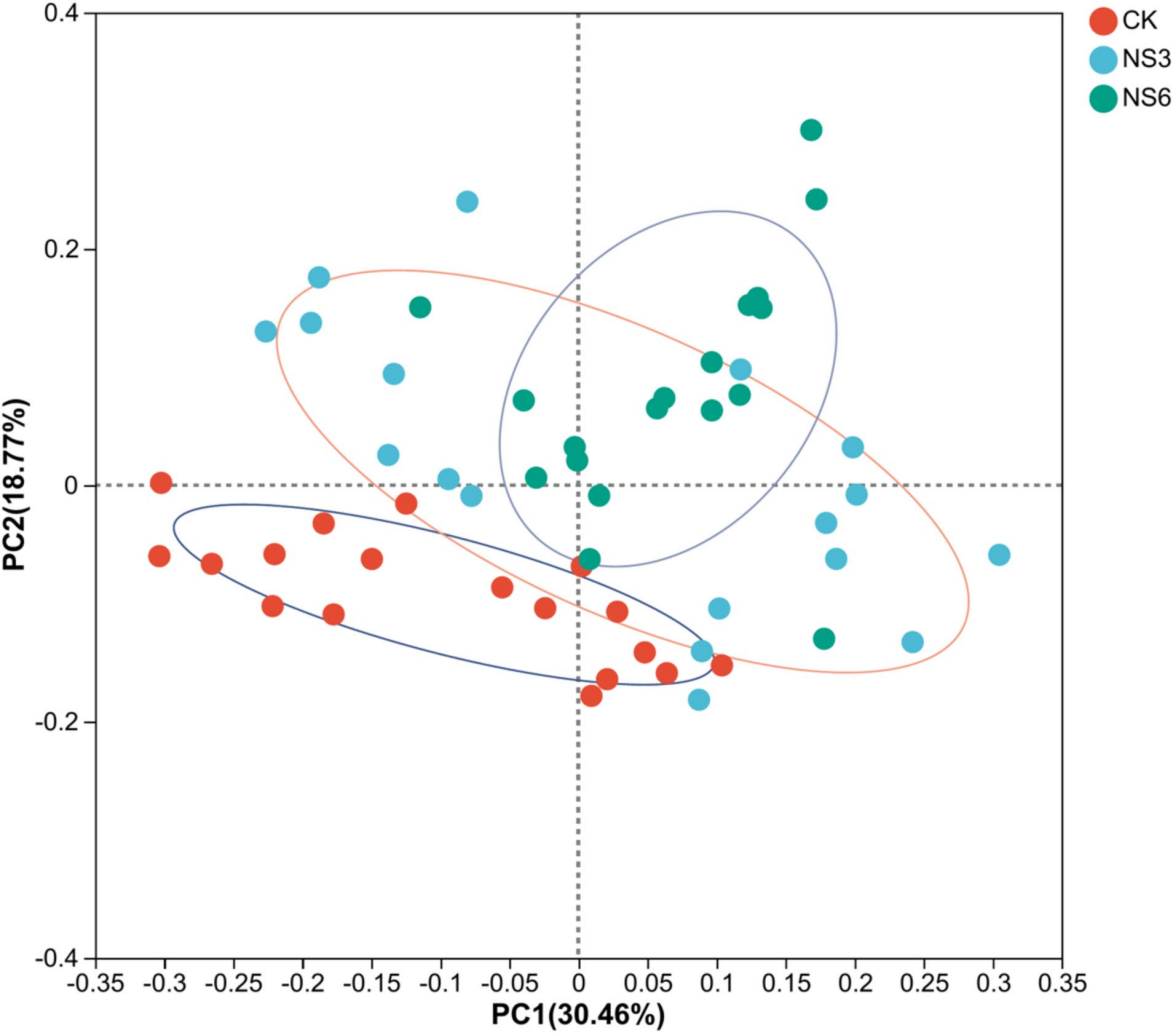
PCoA analysis of bacterial distributions in rhizosphere soil of rice at different treatments. CK, control treatment; NS3, 3‰ NaCl stress treatment; NS6, 6‰ NaCl stress treatment.

PCoA analysis shows that bacterial community structures were distinctly grouped and bacterial significant changed (*p* = 0.001) by control (CK) treatment and NaCl stress treatments. The samples of control (CK) treatment were clustered in PC1; while the samples of NS3 and NS6 treatments with NaCl stress were relatively close in distance, and similarity in community composition (Figure 5).

**Figure 6:**
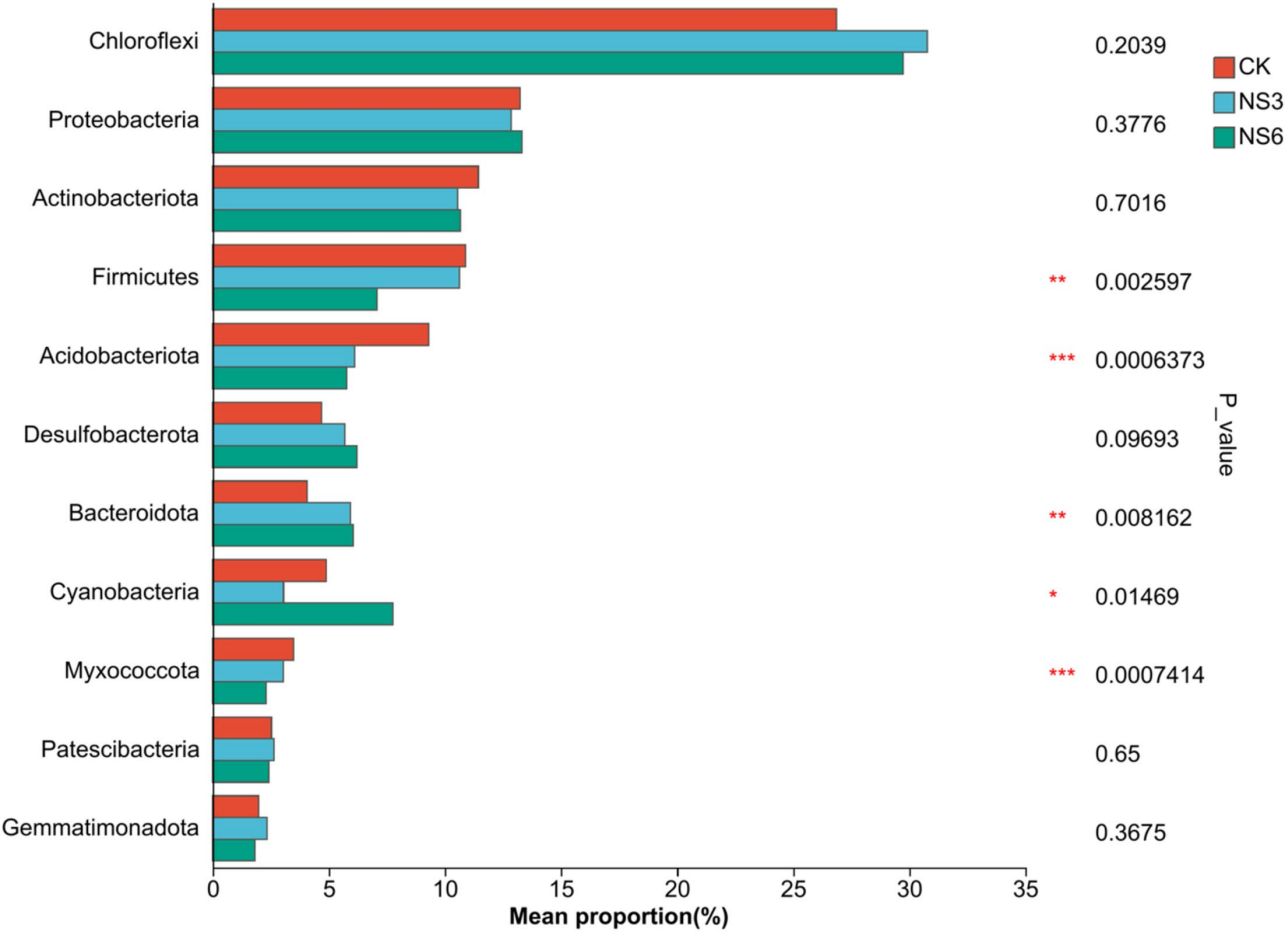
The differences of dominant bacteria groups at the level of phylum. CK, control treatment; NS3, 3‰ NaCl stress treatment; NS6, 6‰ NaCl stress treatment.

To elucidate the differences in bacterial community structures among the various treatments, significance inter group differences of phylum in bacterial communities with relative abundance >2% was examined. The results indicated that the phyla Firmicutes, Acidobacteriota, Bacteroidota, Cyanobacteria and Myxococcota exhibited significant differences among treatments (*p* < 0.01). Specifically, the relative abundances of Firmicutes and Acidobacteriota in the control (CK) treatment were significantly higher than those in the NS3 andNS6 treatment. Similarly, the relative abundances of Myxococcota in the control (CK) treatment and NS3 were significantly higher than those in the NS6 treatment. In contrast, the relative abundances of Bacteroidota were significantly higher in NS6 compared to CK and NS3, and Cyanobacteria was significantly higher in NS6 compared to NS3 (Figure 6). Overall, salt stress reduced the relative abundances of Firmicutes, Acidobacteriota and Myxococcota, while increasing the relative abundances of Bacteroidota and Cyanobacteria.

**Figure 7:**
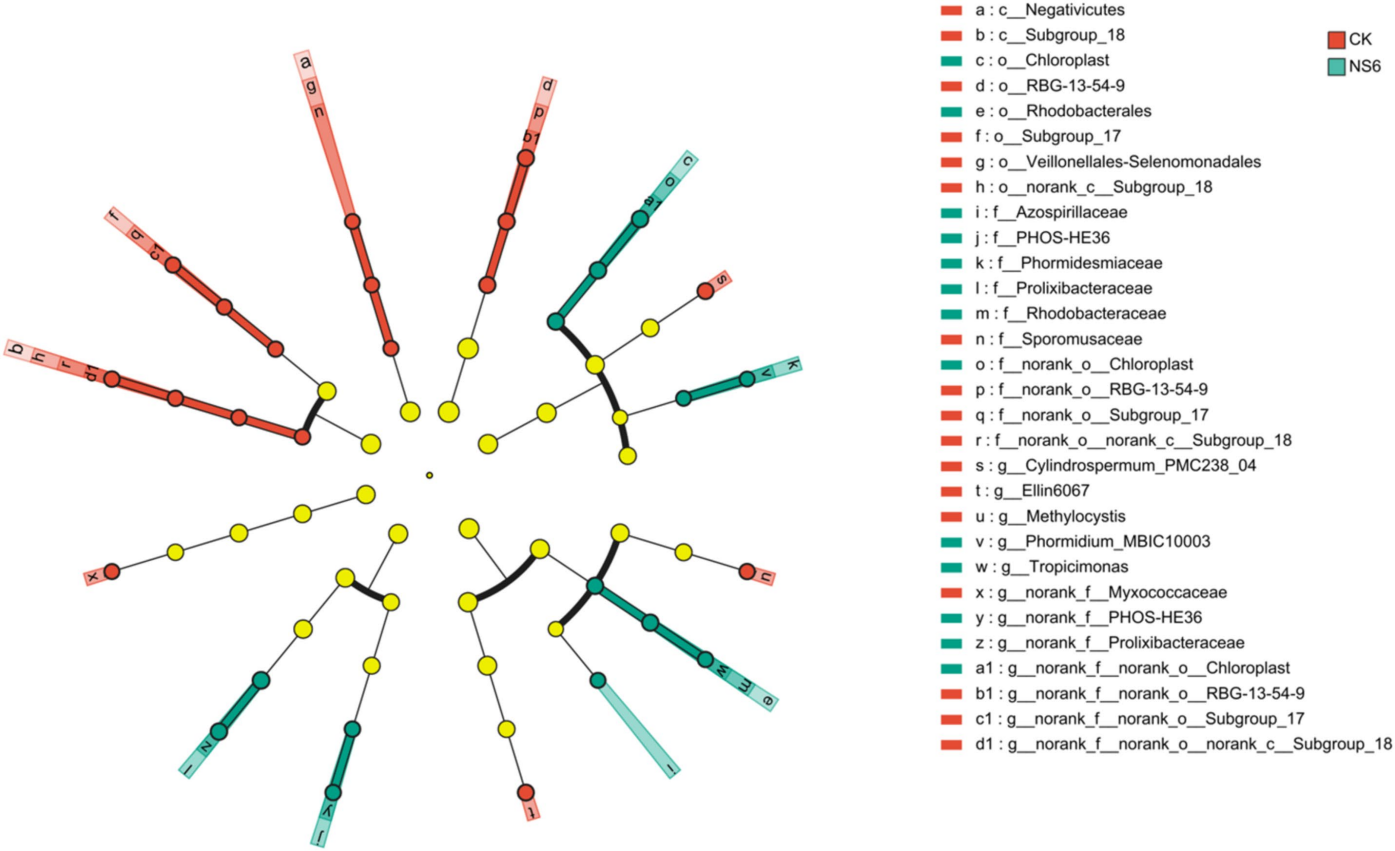
Discriminant analysis of taxa enrichment in each salt stress treatment (LDA scores is 3). CK, control treatment; NS3, 3‰ NaCl stress treatment; NS6, 6‰ NaCl stress treatment.

LEfSe analysis reveald that eight bacterial branch enriched in the rhizosphere soil of rice under CK treatment, Cylindrospermum_PMC238_04, Ellin6067, RBG-13-54-9 (from order to genus), Subgroup_18 (from class to genus), Methylocystis, Sporomusaceae (from class to family), Subgroup_17 (from order to genus) and Myxococcaceae. Six bacterial branch enriched in rhizosphere soil of rice under NS6 treatment, MBIC10003 (from order to family), Tropicimonas (from order to genus), PHOS-HE36 (from family to genus), Prolixibacteraceae (from family to genus), Chloroplast (from order to genus) and Azospirillaceae. But no significant enrichment was detected in rhizosphere soil of rice under NS3 treatment (Figure 7).

### 3.4 Effects of salt stress on bacterial community functions

**Figure 8:**
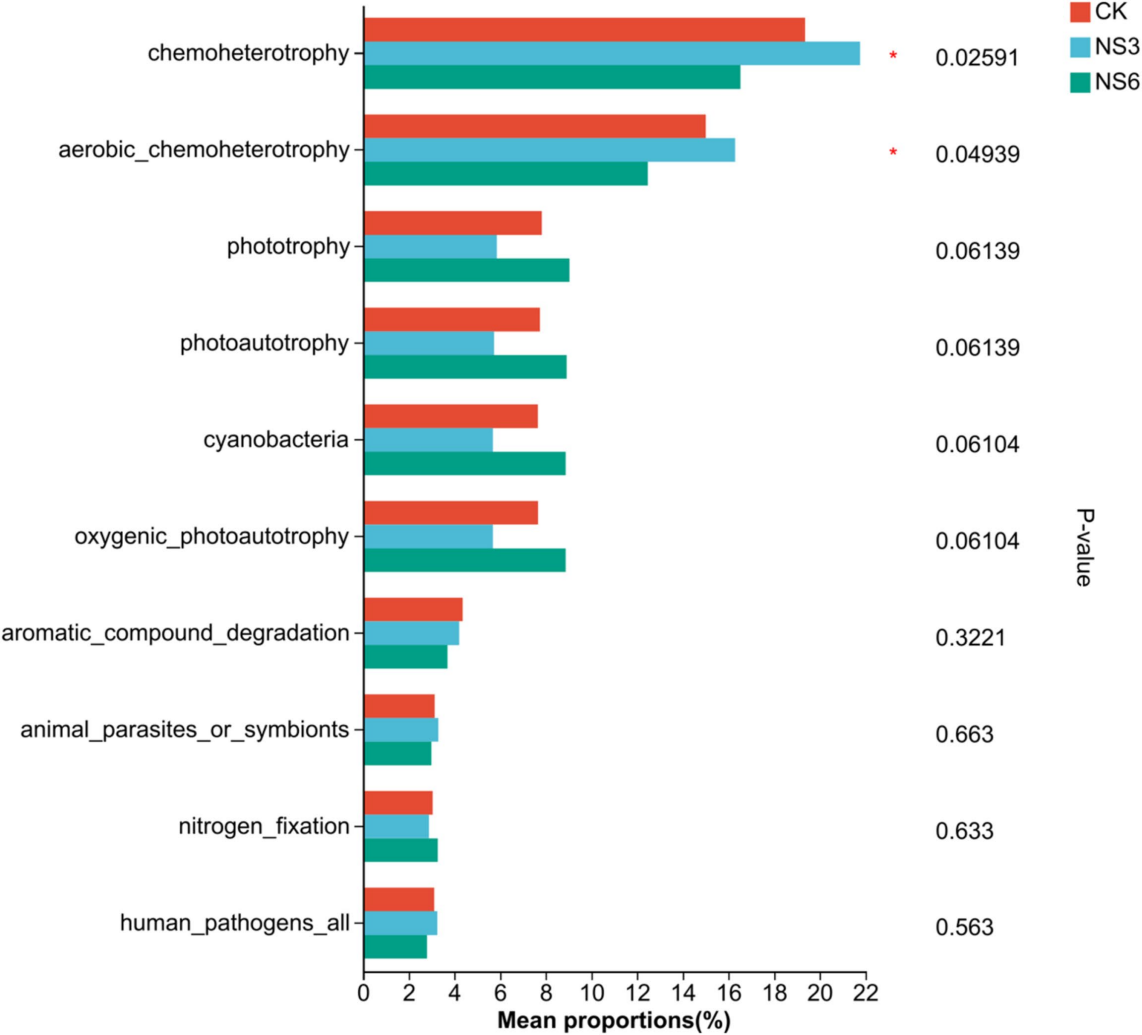
Prediction of bacterial function in rhizosphere soil of rice. CK, control treatment; NS3, 3‰ NaCl stress treatment; NS6, 6‰ NaCl stress treatment.

Bacterial communities in rhizosphere soil of rice are involved in diverse functional pathways, the top 10 functions with total abundance included chemoheterotrophy, aerobic_chemoheterotrophy, phototrophy, photoautotrophy, cyanobacteria, oxygenic_photoautotrophy, aromatic_compound_degradation, animal_parasites_or_symbionts, nitrogen_fixation and human_pathogens_all. Further functional differences were tested among different salt stress treatments. The results showed that there was significantly difference (*p* < 0.01) in the functions of chemoheterotrophy and aerobic_chemoheterotrophy in rhizosphere soil bacterial communities of rice under different salt stress treatments (Figure 8). Among them, chemoheterotrophy and aerobic_chemoheterotrophy were significantly in NS3 treatment higher than NS6 treatment.

## 4 Discussion

Salt stress modulates the microbial diversity and community composition of rhizosphere soil by altering its ecological factors (Rosier et al., 2016; Macabuhay et al., 2022). Previous studies have demonstrated that salt stress diminishes the richness of bacterial communities in the rhizosphere soil (Wu et al., 2010). Additionally, both the richness and diversity of bacterial communities typically decline with increasing salinity levels (Zhao et al., 2020). Under saline-alkali conditions, the growth of rhizosphere microorganisms is inhibited, leading to a significant reduction in community richness (Baumann et al., 2013). In the present study, the richness and diversity indices of bacterial communities subjected to salt stress treatments were markedly lower than those observed in the control (CK) group. Consequently, it can be inferred that salt stress substantially reduces the richness and diversity of bacterial communities in the rhizosphere soil of rice, thereby corroborating the findings of previous research.

In this study, Chloroflexi, Proteobacteria, and Actinobacteria were identified as the primary bacterial phyla in the rhizosphere soil under salt stress, followed by Firmicutes, Acidobacteria, Desulfobacterota, Bacteroidota, and Cyanobacteria. This composition is consistent with findings from most previous studies (Ma and Gong, 2013; Canfora et al., 2014; Zhao et al., 2020; Xu et al., 2022). The prevalence of these bacterial taxa may be attributed to their participation in various nutrient cycling and biogeochemical processes, thereby playing significant roles in ecosystem services. The relative abundance of Chloroflexi was the highest in this study, potentially due to the long-term flooding of paddy soils, which generates methane emissions that are oxidized by aerobic methane-oxidizing bacteria. Chloroflexi are a group of methane-oxidizing bacteria that play a crucial role in methane conversion and carbon cycling in paddy soils. Studies have confirmed that Chloroflexi possess highly diverse metabolic capabilities. In terms of energy metabolism, Chloroflexi can perform both aerobic and anaerobic respiration. Additionally, they can oxidize methane through metabolic pathways, utilize CO₂ as a carbon source for energy production, and degrade refractory carbon compounds under conditions of low nutrient availability (Zhang et al., 2019; Xian et al., 2020). Proteobacteria represent the largest and most diverse bacterial phylum. Many groups within Proteobacteria are capable of nitrogen fixation and adapting to various complex environments. Their extensive phylogenetic and ecological significance makes Proteobacteria the most common microbial group in saline soils (Ladha and Reddy, 2003; Ma and Gong, 2013). The predominance of Proteobacteria in the rhizosphere of most plant species may be attributed to their rapid growth rates (Yang et al., 2017). Actinobacteriota are known to participate in carbon fixation within specific ecological niches (Bryant et al., 2012) and are dominant phyla in secondary saline-alkali soils (Niu et al., 2017). Actinobacteriota are recognized as one of the most prevalent phyla in these environments. These functional microorganisms enriched in the rhizosphere are instrumental in mediating soil nutrient transformation and enhancing the environmental adaptability of microbial communities (Vacheron et al., 2013). Their presence in this study indicates that microbial distributions in similar salt-stressed environments exhibit certain similarities, reinforcing the notion that comparable ecological pressures select for analogous microbial assemblages.

Salinity level control the composition and functional potential of microbial community (Wang et al., 2018). Significant differences demonstrated that salt stress reduced the relative abundances of Firmicutes, Acidobacteriota and Myxococcota, while increasing the relative abundances of Bacteroidota and Cyanobacteria. This indicated that salt stress alters the distribution of bacterial abundances. This is similar to the research results of other scholars. Shi et al. (2021) believe that the relative abundances of Acidobacteriota and Myxococota were significantly decreased in salt-treated soil, while the abundance of Bacteroidota was higher in salt-treated soil. The relative abundances of Bacteroidetes and Cyanobacteria higher of NS6 in our study may be related to its salt tolerance. Research suggests that Bacteroidetes exhibit strong resistance to high-salt environments and are the dominant species in saline-alkali soils (Li et al., 2021). Cyanobacteria has strong salt tolerance, endowed with the potential to curb the negative impacts of salt stress, and to be more efficient in mitigating the deleterious effects caused by the salinity conditions (Padhi et al., 1997; Bello et al., 2021). Chemoheterotrophy and aerobic_chemoheterotrophy were significantly in NS3 treatment higher than NS6 treatment in our study. They are two functions are considered extensive ecosystem functions and are performed by most microorganisms (Rivett and Bell, 2018). This indicates that high salinity level exerted inhibitory effect on chemoheterotrophy and aerobic_chemoheterotrophy of bacterial communities in the rhizosphere soil of rice. But mechanisms influencing bacterial community function in rhizosphere soil of rice under salinization should be further studied.

## 5 Conclusion

In conclusion, this study showed that salt stress changed the diversity, structure and function of bacterial communities in rhizosphere soil of rice. Salt stress reduces the richness and diversity of bacterial communities in rhizosphere soil of rice. The bacterial community was characterized by the dominant bacterial belong to the phyla Chloroflexi, Proteobacteria and Actinobacteria. The relative abundances of Firmicutes, Acidobacteriota and Myxococcota were decreased, while Bacteroidota and Cyanobacteria were increased under salt stress. The functions mainly include chemoheterotrophy, aerobic_chemoheterotrophy, phototrophy etc., chemoheterotrophy and aerobic_chemoheterotrophy were significantly higher NS3 treatment than NS6 treatment of bacterial communities in rhizosphere soil of rice. These results provide a new way for improving the salt tolerance of rice cultivation on saline soil.

## Data Availability

The original contributions presented in the study are included in the article/supplementary material, further inquiries can be directed to the corresponding authors.
